# Exploring the constituent mechanisms of hepatitis: a dynamical systems approach

**DOI:** 10.1093/imammb/dqac013

**Published:** 2022-10-04

**Authors:** Joanne L Dunster, Jonathan M Gibbins, Martin R Nelson

**Affiliations:** Institute for Cardiovascular and Metabolic Research, University of Reading, Reading, RG6 6AS, UK; Institute for Cardiovascular and Metabolic Research, University of Reading, Reading, RG6 6AS, UK; School of Science and Technology, Nottingham Trent University, Nottingham, NG11 8NS, UK

**Keywords:** hepatitis, inflammation, mathematical modelling, bifurcation analysis

## Abstract

Hepatitis is the term used to describe inflammation in the liver. It is associated with a high rate of mortality, but the underlying disease mechanisms are not completely understood and treatment options are limited. We present a mathematical model of hepatitis that captures the complex interactions between hepatocytes (liver cells), hepatic stellate cells (cells in the liver that produce hepatitis-associated fibrosis) and the immune components that mediate inflammation. The model is in the form of a system of ordinary differential equations. We use numerical techniques and bifurcation analysis to characterize and elucidate the physiological mechanisms that dominate liver injury and its outcome to a healthy or unhealthy, chronic state. This study reveals the complex interactions between the multiple cell types and mediators involved in this complex disease and highlights potential problems in targeting inflammation in the liver therapeutically.

## 1. Introduction

Hepatitis is a general term used to describe ongoing, damaging inflammation in the liver that can lead to the development of fibrosis and ultimately liver failure. There are over 100 types of liver disease that have inflammation as a core component. Common causes of an inflamed liver are hepatitis C, a blood borne virus; medications, such as paracetamol; alcohol, which can result in alcohol-related liver disease (ARLD); and obesity and diabetes, which can result in nonalcoholic steatohepatitis (NASH) (Sato *et al.*, 2016). The liver is remarkable in the wide range of functions it performs: it helps convert food into energy; detoxifies chemicals, drugs and toxins; stores vitamins and produces hormones and proteins. Many of these functions require inflammation, the liver being constantly bombarded by a stream of dietary and bacterial products that have inflammatory potential. However, while the liver is generally thought to be a non-immunological organ, it is known that in a healthy liver constant exposure to inflammatory stimuli results in regulation of inflammation (Rius *et al.*, 2012; Koyama & Brenner, 2017). Failure in regulating inflammation can result in a self-perpetuating process where scar tissue is laid down (liver fibrosis) that can progress to cirrhosis and ultimately liver failure Solovyev *et al.* (2013). Given the importance of the liver, it is perhaps surprising that so few treatments for liver disease exist (Koyama & Brenner, 2017), with most existing treatments just focusing on the removal of the stimulus of inflammation so that the liver can heal itself. For example, while treatments (anti-virals) are used to target the virus hepatitis C, which causes liver failure, for liver diseases such as ARLD or NASH the patient would be advised to take preventative measure such as cessation of drinking or a change of diet to limit failure (Sato *et al.*, 2016).

Several authors have proposed models that capture damage to the liver. Remien and coworkers constructed a model that captures the effects of acetaminophen (paracetamol) on the liver’s key cell type, hepatocytes and the downstream consequences on the synthesis of clotting factors (Ramachandran *et al.*, 2012). This model, which has two key enzymes produced by damaged hepatocytes and clotting factor synthesis as outputs, was able to predict laboratory levels of these outputs for patients admitted to hospital following acetaminophen overdose. To study the effects of alcohol on acetaminophen-induced liver damage, this model was later updated to include the mechanisms of alcohol metabolism. The analysis suggested that hepatocyte damage depends on a trade-off between induction and inhibition of key enzymes involved in alcohol metabolism, with the risk of liver damage being increased if acetaminophen is ingested shortly after alcohol, but with simultaneous ingestion resulting in less damage (Ghosh *et al.*, 2021a). A further study by these authors extended this work to investigate ischemic hepatitis and the ability to predict commonly used biomarkers (Ghosh *et al.*, 2021b). Webb and coworkers also focus on the damage to liver by acetaminophen; having constructed a model that captures the main dynamics of acetaminophen metabolism, they used singular perturbation analysis to identify which reactions dominate during the successive stages of metabolism, identifying the critical cutoff between safe and overdose cases (Webb *et al.*, 2015). Friedman and Hao augment their extensive prior work on fibrosis in tissues other than the liver (Hao *et al.*, 2014, 2015) to produce a spatial model of liver fibrosis that captures the interactions between a range of resident cells (such as macrophages, T cells and hepatic stellate cells) and mediators known to regulate fibrosis (Friedman & Hao, 2017). With parameters from their previous work, they provide numerical simulations demonstrating the dependence of scar density in liver fibrosis in terms of the concentrations of the regulators TIMP and HA.

Beyond the context of studying liver tissue specifically, there is a growing repertoire of mathematical models of inflammation in published literature, both in the generic sense (with many mechanisms spanning multiple health scenarios) and in relation to specific tissues or ailments. Generally, many of these models take the approach of simplifying the vast and complex interactions of numerous cell types and inflammatory mediators by constructing models that include only the most important cell types (e.g. neutrophils and macrophages) and generic descriptions of pro- and anti-inflammatory mediators that combine the effects of numerous individual mechanisms. For example, the early work of Lauffenburger and coworkers has examined interactions between motile bacteria, a single type of immune cell and a generic inflammatory mediator, with one focus being upon how the spatial migration of immune cells impacts upon the inflammatory outcome (Ladero *et al.*, 2009; Lauffenburger & Keller, 1979). More recently, mathematical models of inflammation have been published that focus on a wide range of tissues/conditions, including applications in wound healing (Waugh & Sherratt, 2006), spinal chord injury (Smith *et al.*, 2019), sepsis (Cockrell & An, 2018) and many others (although, to date, we are not aware of any such models in the context of liver disease). For a more detailed review of previous inflammation models, see Dunster & Nelson (2022). Of particular relevance to this study are the three previous models of Dunster *et al.* (2014), which describe the interactions of neutrophils (both active and apoptotic), macrophages and pro- and anti-inflammatory mediators in response to generic tissue damage. These ordinary differential equation (ODE) models incorporate a thorough catalogue of interactions and, through dynamical systems analysis and numerical simulations, these models revealed the interactions (and associated parameters) that play the most important role in determining the switch between resolution of damage and chronic injury. In particular, these models identified the phagocytic ability of macrophages and the strength of pro-inflammatory feedback from apoptotic neutrophils as the two most dominant mechanisms in controlling this switch, both of which can be considered as targets for therapeutic interventions. As the models of Dunster *et al.* (2014) form the starting point for the construction of our hepatitis model below, we review the relevant detail of the governing ODEs in the following section. We note that the work of Dunster *et al.* (2014) was also later extended to a spatial setting via partial differential equation (PDE) or agent-based approaches (Bayani *et al.*, 2020a,b) and, while we do not pursue this direction here, the model below is a useful precursor to more complex spatial models of hepatitis progression.

Here, we construct, to our knowledge, the first mathematical model of inflammation as it occurs within, and interacts with the key cell types of, the liver. We start by developing the model (Section 2) before demonstrating its range of outcomes (Section 3). The model is bistable with the healthy response (resolution of damage) being represented by a steady state in which all pro-inflammatory components reach zero, and unhealthy responses (in which liver disease perpetuates) manifesting as either steady states that have positive levels of pro-inflammatory components (with tissue damage also resulting in liver cells being replaced by extracellular matrix (ECM)), or by solutions that oscillate temporally. In Section 4, we use bifurcation analysis to investigate the manner in which variation of key model parameters drives switching between these outcomes, before finally discussing these findings and noting therapeutic implications (in Section 5). Our analysis makes use of the softwares R and Matlab for numerical simulations, and XPP-AUTO for computation of bifurcation diagrams; code to reproduce our numerical simulations and bifurcation analyses can be downloaded from http://github.com/cardiomaths/hepatitisModelling or is available as an archive at time of publication from https://figshare.com/articles/software/hepatitisModelling/19740364/1.

## 2. The mathematical model

**Fig. 1.**
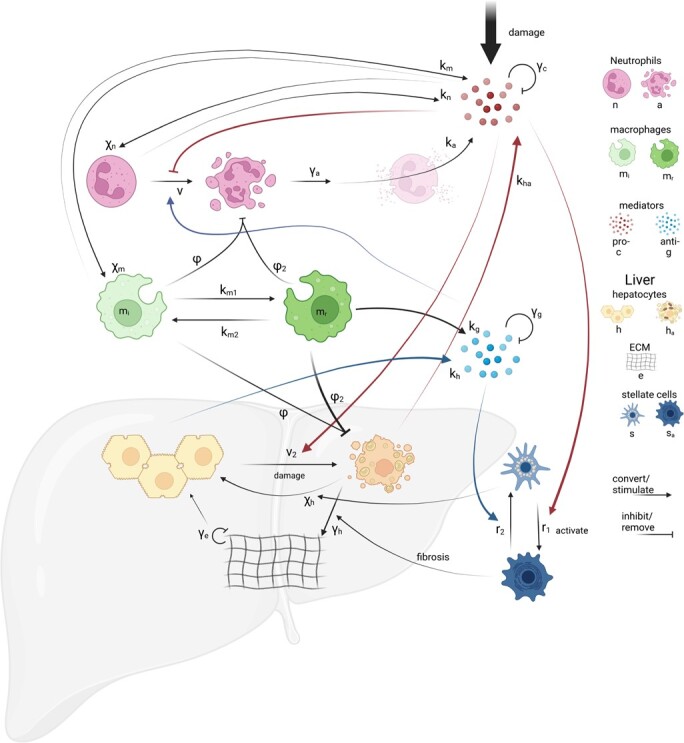
Interactions between the cells and mediators captured in our model of hepatitis. Damage causes a rise in pro-inflammatory mediators, increasing influx of neutrophils and macrophages. Neutrophils (in active or apoptotic form) cause a rise in pro-inflammatory mediators through the release of their toxic content. Macrophages exist in pro- or anti-inflammatory phenotypes which release the associated mediators; both phenotypes remove damaged or dead cells. Pro-inflammatory stimuli damage hepatocytes and activate stellate cells, the later stimulating the production of extracellular matrix (ECM). Dead hepatoctytes are removed by macrophages, being replaced by healthy hepatocytes or ECM, depending on the activation state of stellate cells. Dimensional parameters associated with particular processes are placed next to the relevant arrows and summarized in Table 2.

Our model of hepatitis (depicted in Fig. 1) is intentionally simple: it neglects spatial effects and focuses on the better-known interactions between the liver’s key cell types and those of the acute immune response, neglecting disease-specific details. The inflammatory components of our model are based on earlier work of generic inflammation in a sterile environment (Dunster *et al.*, 2014), these interactions being replicated under damage to the liver (Tanaka & Miyajima, 2016; Torres *et al.*, 2019; Woolbright & Jaeschke, 2017). In this model, populations of active neutrophils ($n$), apoptotic neutrophils ($a$) and macrophages ($m$) interact with pro- and anti-inflammatory mediators ($c$ and $g$, respectively). Active neutrophils arrive (at rate $\chi _n$) in response to pro-inflammatory mediators and die (by apoptosis) at rate $\nu $, both of which are influenced by pro- and anti-inflammatory mediator concentrations. Active neutrophils are a source of pro-inflammatory mediators (at rate $k_n$) but this ceases once neutrophils become apoptotic, until they break down (at rate $\gamma _a$), losing their cell membrane via a process called necrosis that spills their contents into the tissue, increasing pro-inflammatory mediator concentrations (at magnitude $k_a$).

The original model of inflammation in a generic context (Dunster *et al.*, 2014) included just a single population of macrophages; however, it is known that hepatic macrophages form highly heterogeneous populations and, while macrophages consist of a mix of recruited and resident cells, we simplify this and instead focus on just two populations of macrophages that are well known to increase in numbers under hepatitis (Isogawa *et al.*, 2005; Pellicoro *et al.*, 2014; Triantafyllou *et al.*, 2018; Torres *et al.*, 2019; Colino *et al.*, 2020; Wang *et al.*, 2021). Our model incorporates a predominantly pro-inflammatory macrophage phenotype, denoted $m_i$, and an entirely restorative phenotype, denoted $m_r$. The pro-inflammatory macrophages, $m_i$, secrete pro-inflammatory mediators (at rate $k_m$) but also remove dead cells—an anti-inflammatory effect. The restorative macrophages, $m_r$, release anti-inflammatory mediators (at rate $k_g$) and remove dead cells (Pellicoro *et al.*, 2014). The removal of dead cells by the first pro-inflammatory macrophage population is known to promote a switch in macrophage phenotype (modelled with rate $k_{m1}$) to the later restorative population which have a greater phagocytic capacity (Pellicoro *et al.*, 2014; Tang *et al.*, 2021). Following the original model, we include a parameter $\phi $ to represent the rate of phagocytosis of dead cells and introduce a further dimensionless scaling parameter $\phi _2$ to capture the relative phagocyctic ability of $m_r$ macrophages over $m_i$. The reversal of macrophage phenotypes is controversial; some authors believing that opposing phenotypes derive from populations of monocytes. We follow Pellicoro *et al.* (2014) and include the regression from restorative to pro-inflammatory phenotype, at rate $k_{m2}$. Macrophages are slow to die and we follow our original model and include this at rate $\gamma _{m}$ but augment this by including the influence of restorative macrophages on the rate that pro-inflammatory macrophages die (with associated rate parameter $\gamma _{m2}$) (Robinson *et al.*, 2016). These modelling assumptions result in the following equations for the inflammatory cells and mediators:

(1a) \begin{align*} \frac{dn}{dt} &= \chi_n\frac{c}{1+\frac{g}{\beta_{gc}}} - \nu\frac{1+\frac{g}{\beta_g}}{1+\frac{c}{\beta_c}} n,\\\frac{da}{dt} &= \nu\frac{1+\frac{g}{\beta_g}}{1+\frac{c}{\beta_c}} n - \gamma_a \,a - \phi\, a\, (m_i + \phi_2\, m_r),\tag{(1b)}\\\frac{dm_i}{dt} &= \chi_m\, c - k_{m1}\, \phi\, a\, m_i + k_{m2}\, m_r - \gamma_m\, m_i\left(1+\gamma_{m2}\,m_r\right),\tag{(1c)}\\ \frac{dm_r}{dt} &= k_{m1}\, \phi\, a\, m_i - k_{m2}\, m_r - \gamma_m\, m_r,\tag{(1d)}\\\frac{dc}{dt} &= k_a\,\gamma_a \frac{a^2}{\beta_a^2 + a^2} + k_n\frac{n^2}{\beta_n^2+n^2} + k_{m}\, m_i - \gamma_c\, c,\tag{(1e)}\\\frac{dg}{dt} &= k_g\, m_r - \gamma_g\, g.\tag{(1f)} \end{align*}

The liver is comprised predominantly of hepatocytes, approximately 80% by weight, and they are responsible for performing the majority of the liver’s functions (Duarte *et al.*, 2015). Under inflammatory conditions hepatocytes are vulnerable to damage and the liver accumulates excessive ECM, leading to a deterioration in liver function and, if not resolved, ongoing inflammation can lead to cirrhosis and ultimately liver failure (Bataller & Brenner, 2005; Gressner & Weiskirchen, 2006; Dirchwolf & Ruf, 2015; Khailaie *et al.*, 2020). We assume that the structure of the liver tissue itself is fundamentally composed of hepatocytes (both active and damaged, $h$ and $h_a$, respectively) and ECM (represented by variable $e$). We prescribe the following equations for these, which are configured such that the quantity $h_T=h+h_a+e$ is conserved:

(1g) \begin{align*} \frac{dh}{dt} &= \chi_h \,\phi \frac{s}{s_T} h_a (m_i + \phi_2 m_r) + \gamma_e\, e - \nu_2\, h\, c + \gamma_h\, h_a \frac{s}{s_T},\\\frac{dh_a}{dt} &= \nu_2\, h\, c - \chi_h\,\phi\, h_a (m_i + \phi_2\, m_r) - \gamma_h h_a,\tag{(1h)}\\\frac{de}{dt} &= \chi_h\, \phi\, \frac{s_a}{s_T} h_a (m_i + \phi_2\, m_r)- \gamma_e\, e + \gamma_h\, h_a \frac{s_a}{s_T}.\tag{(1i)} \end{align*}

Above, pro-inflammatory mediators increase hepatocyte damage (at rate $\nu _2$), damaged hepatocytes are removed by macrophages (with rate parameters $\chi _h$, $\phi $ and $\phi _2$) and hepatocytes lyse at rate $\gamma _h$. As hepatocytes are lost, they are replaced with either new hepatocytes or ECM, which itself lyses at rate $\gamma _e$ being replaced by hepatocytes. The replacement of hepatocytes by ECM is promoted by active hepatic stellate cells. These exist in liver capillaries in a quiescent state ($s$) but, under the influence of pro-inflammatory mediators can transform (with rate $r_1$) to a myofibroblast-like phenotype ($s_a$), a process termed activation (Liu *et al.*, 2021). They can also return to their original state in the presence of anti-inflammatory mediators (with rate $r_2$) (Liu *et al.*, 2021; Wynn & Barron, 2010). The equations for stellate cells, once again configured such that the total population of stellate cells ($s+s_a=s_T$) are conserved, are as follows:

(1j) \begin{align*} \frac{ds}{dt} &= r_2 s_a \left(1 + \frac{g}{\beta_{gc}}\right) - r_1 s c, \end{align*}

(1k) \begin{align*} \frac{ds_a}{dt} &= r_1 s\, c - r_2 s_a \left(1 + \frac{g}{\beta_{gc}}\right). \end{align*}

Finally, we modify the equations for inflammatory mediators (1e,1f) to reflect positive and negative feedbacks from hepatocytes. When the fraction of active hepatocytes is high, we expect strong production of anti-inflammatory mediators, the rate of production of which we denote as $k_h$ (per cell). Similarly, if the proportion of damaged hepatocytes is high, we expect production of pro-inflammatory mediators at rate $k_{ha}$ per cell (Lauffenburger & Kennedy, 1983). Accordingly, we modify (1e,1f) as follows:

(1l) \begin{align*} \frac{dc}{dt} &= k_a\,\gamma_a \frac{a^2}{\beta_a^2 + a^2} + k_n\frac{n^2}{\beta_n^2+n^2}+ k_{ha}\, \frac{h_a}{h_T} + k_{m}\, m_i - \gamma_c\, c,\tag{(1l)}\\\frac{dg}{dt} &= k_g\, m_r + k_{h}\, \frac{h}{h_T} - \gamma_g\, g.\tag{(1m)} \end{align*}

We close the system (1) by imposing initial conditions $n(0)=a(0)=m_i(0)=m_r(0)=g(0)=h_a(0)=e(0)=s_a(0)=0$, $h(0)=h_T$, $s(0)=s_T$ and $c(0)=c_0$, i.e. we assume that at $t=0$ liver tissue is healthy, being composed of a population of active hepatocytes ($h_T$) and quiescent stellate cells ($s_T$) and damage is stimulated by a burst of pro-inflammatory mediators of concentration $c_0$. A network diagram representing the events incorporated into this system of equations is shown in Fig. 1 while a summary of the model’s variables and parameters are given in Tables 1 and 2, respectively.

**Table 1:** Summary of the dependent variables appearing in our model.

**Variable**	**Description**	**Units**
$n$	Neutrophils	cell mm$^-3$
$a$	Apoptotic neutrophils	cell mm$^-3$
$m_i$	Pro-inflammatory macrophages	cell mm$^-3$
$m_r$	Restorative (anti-inflammatory) macrophages	cell mm$^-3$
$c$	Pro-inflammatory mediator	pg mm$^-3$
$g$	Anti-inflammatory mediator	pg mm$^-3$
$h$	Hepatocytes	cell mm$^{-3}$
$h_a$	Damaged hepatocytes	cell mm$^{-3}$
$e$	ECM	cell mm$^{-3}$
$s$	Stellate cells (quiescent)	cell mm$^{-3}$
$s_a$	Active stellate cells	cell mm$^{-3}$

**Table 2:** Summary of the dimensional parameters that appear in (1)-indicate nondimensional unit of measure.

**Parameter**	**Description**	**Units**
Inflammatory components (from Dunster *et al.*, 2014)		
$\phi $	Removal of dead cells by macrophages	cell$^{-1}$ mm$^{3}$ day$^{-1}$
$\nu $	Rate of neutrophil apoptosis	day$^{-1}$
$\chi _n$	Rate of arrival of neutrophils	cell pg$^{-1}$ day$^{-1}$
$\chi _m$	Rate of arrival of macrophages	cell pg$^{-1}$ day$^{-1}$
$\gamma _a$	Rate of apoptotic neutrophil necrosis	day$^{-1}$
$\gamma _c$	Rate of decay of pro-inflammatory mediator	day$^{-1}$
$\gamma _g$	Rate of decay of anti-inflammatory mediator	day$^{-1}$
$\gamma _m$	Rate that macrophages leave/die	day$^{-1}$
$k_a$	Production of pro-inflammatory mediators by apoptotic neutrophils	pg mm$^{-3}$
$k_g$	Production of anti-inflammatory mediators by $m_r$ macrophages	pg cell$^{-1}$ day$^{-1}$
$k_n$	Production of pro-inflammatory mediators by active neutrophils	pg mm$^{-3}$ day$^{-1}$
$\beta _a$	Saturation constant	cell mm$^{-3}$
$\beta _n$	Saturation constant	cell mm$^{-3}$
$\beta _c$	Saturation constant	pg mm$^{-3}$
$\beta _g$	Saturation constant	pg mm$^{-3}$
$\beta _{gc}$	Saturation constant	pg mm$^{-3}$
Inflammatory components (newly introduced)		
$\phi _2$	Increased rate of phagocytosis due to $m_r$ macrophages	—
$k_{m}$	Rate of production of pro-inflammatory mediators by $m_i$ macrophages	pg cell$^{-1}$ day$^{-1}$
$k_{m1}$	Rate of macrophage phenotype switching: $m_i$ to $m_r$	—
$k_{m2}$	Rate of macrophage phenotype switching: $m_r$ to $m_i$	day$^{-1}$
$\gamma _{m2}$	Increase in loss of $m_i$ macrophages, under the influence of $m_r$	cell$^{-1}$ mm$^{3}$
Hepatocytes/ECM		
$\nu _2$	Rate of hepatocyte activation/apoptosis	pg$^{-1}$ mm$^{3}$ day$^{-1}$
$\chi _h$	Proliferation of hepatocytes	—
$\gamma _e$	Rate of ECM decay	day$^{-1}$
$\gamma _h$	Rate of hepatocyte death/leave	day$^{-1}$
$k_{ha}$	Rate of production of inflammatory mediators	pg mm$^{-3}$ day$^{-1}$
$k_{h}$	Rate of production of anti-inflammatory mediators	pg mm$^{-3}$ day$^{-1}$
$h_T$	Size of hepatocyte population	cell mm$^{-3}$
Stellate cells		
$r_1$	Rate of stellate cell activation	pg$^{-1}$ mm$^{3}$ day$^{-1}$
$r_2$	Rate at which active stellate cells become quiescent	day$^{-1}$
$s_T$	Size of stellate cell population	cell mm$^{-3}$

This system (1) is nondimensionalized, using tildes to represent dimensionless quantities. In a similar manner to previous work (Dunster *et al.*, 2014) time is scaled so that $\tilde {t} = \gamma _c t$ and the variables representing immune cells and mediators are scaled as follows:

$$ \begin{align*} c=k_{a}\tilde{c}\text{,}\qquad n=\frac{\chi_n\,k_{a}}{\gamma_{c}}\tilde{n}\text{,}\qquad a=\frac{\chi_n\,k_{a}}{\gamma_{c}}\tilde{a}\text{,}\qquad m_i=\frac{\chi_m k_{a}}{\gamma_{c}}\tilde{m_i}\text{,} \qquad m_r=\frac{\chi_m k_{a}}{\gamma_{c}}\tilde{m_r}\text{,}\qquad g=\beta_{gc}\tilde{g}\text{.} \end{align*}$$

The remaining variables, representing hepatocytes, stellate cells and ECM, are scaled such that

$$ \begin{align*} h=\frac{h_Tk_a \gamma_c}{k_{ha}}\tilde{h}\text{,}\qquad h_a=\frac{h_Tk_a \gamma_c}{k_{ha}}\tilde{h_a}\text{,}\qquad e=\frac{h_Tk_a \gamma_c}{k_{ha}}\tilde{e}\text{,}\qquad s_=s_T\tilde{s}\text{,}\qquad s_a=s_T\tilde{s_a}\text{.} \end{align*}$$

Under the above rescalings, (1) transforms to give the following system of eleven dimensionless equations, where tildes are dropped for clarity:

(2a) \begin{align*} \frac{dn}{dt} &= \frac{c}{1+g} - \nu\frac{1+\frac{g}{\beta_g}}{1+\frac{c}{\beta_c}} n,\\\frac{da}{dt} &= \nu\frac{1+\frac{g}{\beta_g}}{1+\frac{c}{\beta_c}} n - \gamma_a a - \phi\, a\, (m_i + \phi_2\, m_r),\tag{(2b)}\\\frac{dm_i}{dt} &= c - k_{m1}\, \phi\, a\, m_i + k_{m2}\, m_r - \gamma_m\, m_i\left(1+\gamma_{m2}\,m_r\right),\tag{(2c)}\\\frac{dm_r}{dt} &= k_{m1}\, \phi\, a\, m_i - k_{m2}\, m_r - \gamma_m\, m_r,\tag{(2d)}\\\frac{dc}{dt} &= \gamma_a \frac{a^2}{\beta_a^2 + a^2} + k_n\frac{n^2}{\beta_n^2+n^2}+ h_a + k_{m}\, m_i - c,\tag{(2e)}\\\frac{dg}{dt} &= k_g\, m_r + k_{h}\, h - \gamma_g\, g,\tag{(2f)}\\\frac{dh}{dt} &= \chi_h\phi \, s \, h_a (m_i + \phi_2 \, m_r) + \gamma_e \, e - \nu_2 \, h \, c + \gamma_h \, h_a \, s,\tag{(2g)}\\\frac{dh_a}{dt} &= \nu_2 \, h \, c - \chi_h\phi \, h_a (m_i + \phi_2 m_r) - \gamma_h \, h_a,\tag{(2h)}\\\frac{de}{dt} &= \chi_h\phi \,s_a \,h_a (m_i + \phi_2 m_r)- \gamma_e \,e + \gamma_h\, h_a\, s_a,\tag{(2i)}\\\frac{ds}{dt} &= r_2 \,s_a (1 + g) - r_1\, s c,\tag{(2j)}\\\frac{ds_a}{dt} &= r_1\, s\, c - r_2\, s_a (1 + g),\tag{(2k)} \end{align*}

which depends upon the following new dimensionless parameters:

(3a) \begin{gather*} \tilde{\phi}=\frac{\phi\chi_m k_a}{\gamma_c^2}\text{,}\quad \tilde{\nu}=\frac{\nu}{\gamma_c},\quad \tilde{\beta_g}=\frac{\beta_g}{\beta_{gc}},\quad \tilde{\beta_c}=\frac{\beta_c}{k_a},\quad \tilde{\beta_a}=\frac{\beta_a \gamma_c}{\chi_n k_a}\text{,}\quad \tilde{\beta_n}=\frac{\beta_n \gamma_c}{\chi_n k_a}\text{,}\quad \tilde{\gamma}_{a}=\frac{\gamma_{a}}{\gamma_c},\quad \tilde{\gamma}_{e}=\frac{\gamma_{e}}{\gamma_c}, \end{gather*}

(3b) \begin{gather*} \tilde{\gamma}_{h}=\frac{\gamma_{h}}{\gamma_c},\quad \tilde{\gamma}_{g}=\frac{\gamma_{g}}{\gamma_c}\text{,}\quad \tilde{\gamma}_{m}=\frac{\gamma_{m}}{\gamma_{c}},\quad \tilde{\gamma}_{m2}=\frac{\gamma_{m2}\chi_m k_a}{\gamma_c},\quad \tilde{k}_{m1}=\frac{k_{m1}\chi_n}{\chi_m},\quad \tilde{k}_{m2}=\frac{k_{m2}}{\gamma_c},\quad \tilde{k}_{m}=\frac{k_{m}\chi_m}{\gamma_c^2}, \end{gather*}

(3c) \begin{gather*} \tilde{k}_n=\frac{k_n}{k_a\gamma_c},\quad \tilde{k}_g=\frac{k_g \chi_m k_a}{\beta_{gc}\gamma_c^2},\quad \tilde{k}_{h}=\frac{k_{h}k_{a}}{k_{ha}\beta_{gc}},\quad \tilde{r}_1=\frac{r_1 k_a}{\gamma_c},\quad \tilde{r}_2=\frac{r_2}{\gamma_c},\quad \tilde{\nu}_2=\frac{\nu_2k_a}{\gamma_c}. \end{gather*}

The initial conditions transform to give $n(0)=a(0)=m_i(0)=m_r(0)=g(0)=h_a(0)=e(0)=s_a(0)=0$, $h(0)=1$, $s(0)=1$ and $c(0)=c_0$, with $c_0$ being varied to reflect the severity of the stimulus.

The nondimensional parameter groupings that appear in (2) are summarized in Table 3. We note that obtaining precise values for many of the dimensional parameters is difficult, there being a lack of liver-specific reaction rates in the literature. We therefore carry default baseline values for those parameters appearing in the left-hand side of Table 3 from the previous works of Dunster *et al.* (2014) and Bayani *et al.* (2020a). The rate of transition of macrophages from $m_i$ to $m_r$ phenotypes is thought to be an order of magnitude greater than the reverse process (Tang *et al.*, 2021). We therefore set $k_{m1}=30$ and $k_{m2}=0.3$ as our default values for these parameters. The remaining parameters are difficult to infer accurately from the current literature. We expect that the pro-inflammatory feedbacks that appear on the right-hand side of (2e) are of similar magnitudes, in general. While the feedbacks from active/apoptotic neutrophils are known to saturate as these populations increase in size (see Dunster *et al.*, 2014, and the references therein), this is not the case with the macrophage feedback parameterized by $k_m$. Since the population of inflammatory macrophages is, in general, much larger than $\mathcal {O}(1)$ in our model, we expect $k_m$ to be orders of magnitude smaller than other pro-inflammatory rate parameters. We hence set $k_m=0.0001$ in order to preserve a balance between the scales of neutrophil, hepatocyte and macrophage feedbacks in (2e). (Numerical investigations indicate that such small choices of $k_m$ are also necessary to preserve the essential biological bistability in our model, as we will see below.) Intuitively, we expect $\gamma _{m2}\ll 1$, and hence set $\gamma _{m2}=0.01$, and on the basis that we expect anti-inflammatory mediator production by macrophages and hepatocytes to occur at similar rates, we set $\gamma _h=0.1$. Since we expect turnover of hepatocytes (and ECM) to be slower than the rate of apoptotic neutrophil lysis ($\gamma _a$), but faster than the rate of loss of macrophages ($\gamma _m$), we set $\gamma _h=\gamma _e=0.1$. The parameter $\chi _h$ effectively represents a ratio between how quickly macrophages remove apoptotic neutrophils (which occurs at rate proportional to $\phi $) and how quickly macrophages remove active hepatocytes (which occurs at rate proportional to $\chi _h\phi $). On the assumption that these processes are in fact of similar rates, we set $\chi _h=1$ by default. We assume that switching between stellate cell phenotypes $s$ and $s_a$ occurs at similar rates in both directions, and hence set $r_1=r_2=1$ by default. We examine the model’s sensitivity to our choices of these parameter values below.

**Table 3:** Default dimensionless parameter values used in our simulations. The parameters in the left-hand columns are carried directly from Bayani *et al.* (2020a); Dunster *et al.* (2014); those in the right-hand columns are estimated as described in the text. See Table 2 for definitions of the corresponding dimensional parameters.

**Parameter**	**Baseline values**	**Parameter**	**Baseline values**
$\nu $	0.1	$\nu _2$	0.01
$\phi $	0.1	$\phi _2$	10
$\gamma _a$	1	$\chi _h$	1
$\gamma _m$	0.01	$\gamma _e$	0.1
$\gamma _g$	1	$\gamma _h$	0.1
$k_n$	0.01	$\gamma _{m2}$	0.01
$k_g$	0.1	$k_{h}$	0.1
$\beta _a$	0.1	$k_m$	0.0001
$\beta _n$	0.1	$k_{m1}$	30
$\beta _c$	0.12	$k_{m2}$	0.3
$\beta _g$	0.01	$r_1$	1
		$r_2$	1

## 3. Permissible outcomes

**Fig. 2.**
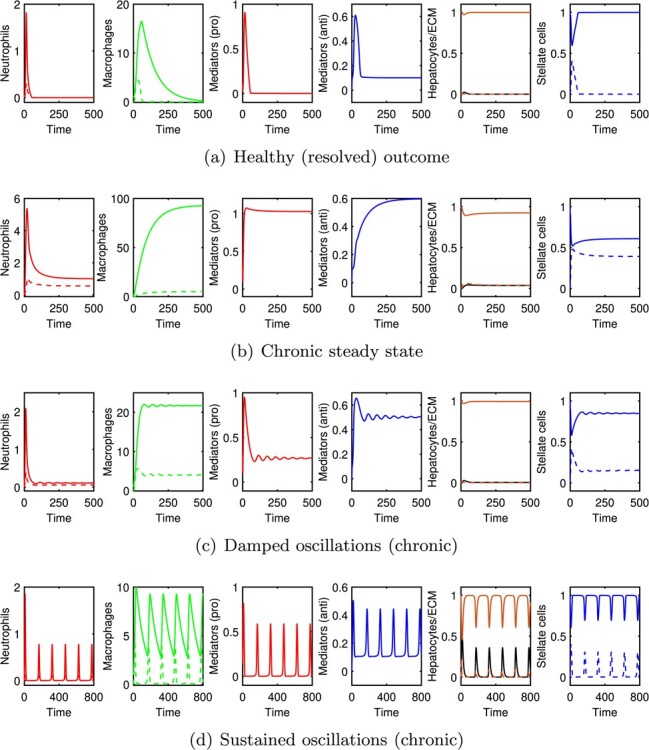
Typical outcomes produced by the model of (2). Solid/dashed lines respectively show: active/apoptotic neutrophils; $m_i$/$m_r$ macrophages; healthy/damaged hepatocytes (with ECM in black); quiescent/active stellate cells. Parameters and initial conditions used are as follows: (a) $\phi =0.045$, $c_0=0.1$, (b) $\phi =0.001$, $c_0=0.3$, (c) $\phi =0.0325$, $c_0=0.3$, (d) $\nu _2=0.5$, $c_0=0.3$); unspecified parameter values are as given in Table 3.

The model of (2) has the potential to exhibit a range of distinct outcomes, with the switch between these outcomes being driven by the tandem effect of varying initial conditions (i.e. the strength of the damage stimulus) and/or variations in the parameter values that govern the strength of the individual interactions. Some of these outcomes are illustrated in Fig. 2. In Fig. 2(a), we illustrate a healthy (fully resolved) outcome in which (after an initial peak in the concentrations of some inflammatory components) the levels of all pro-inflammatory components ultimately settle at zero. This outcome corresponds to a steady state solution of (2) with $h=s=1$, $g=k_h/\gamma _g$ and all other variables equal to zero. (We investigate the stability of this steady state below.) Alternatively, for some parameter choices, the model can exhibit various types of chronic response: either chronic steady states, with pro-inflammatory variables taking positive values (Fig. 2(b and c)); or sustained oscillations as shown in Figure 2(d). Depending on our choice of parameters, the model may be bistable, with both healthy and chronic outcomes permissible and determined by our choice of initial conditions, or monostable, with either a healthy or chronic outcome being guaranteed for all choices of initial condition, the other being unstable.

Computing the stability of chronic states is intractable from an analytic perspective in general; we hence perform this task numerically via bifurcation analysis using the software XPP-AUTO below. However, we are able to compute the stability of the trivial (healthy) steady state analytically, by constructing the Jacobian matrix corresponding to the system of (2), evaluating it at these steady state values and requiring that all the eigenvalues of the resulting matrix have negative real part for stability. For ease of calculation, we reduce the system to nine equations, noting that the equations for $e$ and $s_a$ can be removed and these variables replaced by the algebraic expressions $e=1-h-h_a$ and $s_a=1-s$ due to conservation. The reduced system provides nine eigenvalues, six of which are negative for all choices of parameters. The remaining three eigenvalues ($\lambda $) are given by solutions to the cubic equation

(4) \begin{align*}& \lambda^3+\underbrace{\left(1+\gamma_h+\gamma_m\right)}_{a_2}\lambda^2+\underbrace{\left(\gamma_h+\gamma_m-k_m-\nu_2+\gamma_h\gamma_m\right)}_{a_1}\lambda+\underbrace{\left(\gamma_h\gamma_m-\gamma_h k_m -\gamma_m\nu_2\right)}_{a_0}=0. \end{align*}

Applying the Routh–Hurwitz stability condition, we ascertain that (4) has three roots of negative real part provided that $a_0,a_1,a_2>0$ and $a_1a_2>a_0$. Expanding these conditions provides the following restrictions on parameters, which must all be satisfied in order for a healthy (resolved) outcome to be permissible:

(5a) \begin{gather*} \left(\gamma_h+\gamma_m\right)^2+\left(\gamma_h+\gamma_m\right)\left(\gamma_h\gamma_m+1\right)>k_m\left(\gamma_m+1\right)+\nu_2\left(\gamma_h+1\right), \end{gather*}

(5b) \begin{gather*} \gamma_h+\gamma_m+\gamma_h\gamma_m>k_m+\nu_2, \end{gather*}

(5c) \begin{gather*} \gamma_m\left(1-\frac{\nu_2}{\gamma_h}\right)>k_m. \end{gather*}

For the parameters used here, at least, satisfying the final of these inequalities is sufficient to ensure that the other two are satisfied also. Thus, the potential for a healthy (resolved) outcome is determined by a delicate balance between the pro-inflammatory contributions of macrophages ($k_m$), the susceptibility to damage of hepatocytes ($\nu _2$) and the rates of removal of these cells ($\gamma _m$, $\gamma _h$, respectively). For sufficiently large choices of the parameter capturing susceptibility of hepatocytes to damage ($\nu _2$), in particular, the model is guaranteed to yield a chronic outcome.

Below, we investigate the broader role of each of the key parameters in our model in determining the existence and stability of the various solutions discussed above, focusing in particular upon mechanisms that are (or could be) potential therapeutic targets.

## 4. Bifurcation analysis

Here, we examine the behaviour and outcomes of the model as we vary parameters. Since there are 23 nondimensional parameters in this model, we focus on those parameters that have previously been shown to be key to controlling the switch between healthy and chronic outcomes or represent mechanisms that are under investigation as therapeutic targets for hepatitis, such as the inhibition or promotion of mediators and the prevention of damage to hepatocytes (Koyama & Brenner, 2017). Throughout the following sections, we compute bifurcation diagrams via the numerical continuation software XPP-AUTO. (For an accessible introduction to this software, see Gandy & Nelson, 2022.)

### 4.1 Varying rates of neutrophil apoptosis and macrophage phagocytosis

The previous works of Dunster *et al.* (2014) and Bayani *et al.* (2020a) identified the rates of neutrophil apoptosis ($\nu $) and the removal of apoptotic neutrophils due to phagocytic action by macrophages ($\phi $) as key drivers in determining the boundary between bistability and guaranteed resolution of inflammatory damage. We, here, examine the role of these two parameters in our model.

**Fig. 3.**
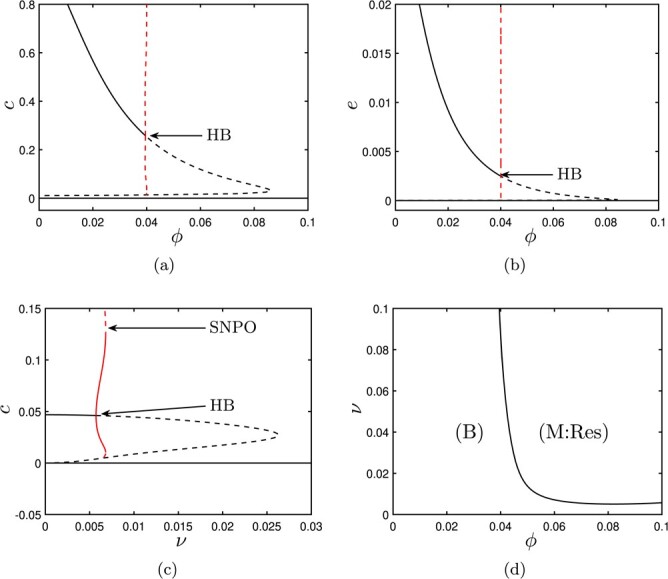
Bifurcation diagrams for the system (2) and the parameters values given in Table 3. In (a–c), solid (*resp.* dashed) black lines represent stable (*resp.* unstable) fixed points; solid (*resp.* dashed) red lines represent stable (*resp.* unstable) periodic orbits. (a,b) Bifurcation diagrams for values in Table 3, showing $c$ and $e$ for varying $\phi $. For small values of $\phi $, the model is bistable, with both healthy and chronic outcomes permissible. As $\phi $ is increased, the chronic steady state becomes unstable via a subcritical Hopf bifurcation (HB) at $\phi =\phi _{HB}\simeq 0.04$, further increases and the system becomes monostable. (c) Bifurcation diagram for values in Table 3 varying $\nu $. The model is bistable for values of $\nu <\nu _{HB}\simeq 0.006$, at which point the chronic steady state becomes unstable via a supercritical Hopf bifurcation, giving rise to stable periodic orbits within a narrow band of $\nu $–values, these being eliminated via a saddle node of periodic orbits (SNPO) as $\nu $ increases. In (d), we show the position of the Hopf bifurcation in $(\phi ,\nu )$–space, identifying regions of bistability (B) and monostability with guaranteed resolution of damage (M:Res).

In Fig. 3, we construct bifurcation diagrams that summarize the existence and stability of our various solutions for varying choices of either $\phi $ or $\nu $ (holding all other parameters fixed at the values given in Table 3). In Fig. 3 (and all subsequent bifurcation diagrams), solid/dashed lines demark stable/unstable solutions respectively, with black lines representing steady states and red lines showing the amplitudes of periodic solutions. Unstable solutions are interesting only from a theoretical (dynamical systems) perspective and will not be observed in simulations; it is only the stable solutions that correspond to realistic, observable outcomes. Figure 3(a) illustrates how the stability of our solutions evolves as the parameter for macrophage phagocytosis ($\phi $) is varied. Here, we see that the healthy steady state (with $c=0$) is stable for all choices of $\phi $ (since the parameter values of Table 3 satisfy the stability condition of (5c) regardless of $\phi $). The healthy (resolved) outcome is therefore always permissible for these parameter values. For low values of $\phi $, a chronic steady state (with $c>0$) exists and is also stable; the model is therefore bistable for these choices of $\phi $, with the observed outcome being determined by the magnitude of the damage stimulus imposed by the initial conditions. As $\phi $ is increased through $\phi =\phi _{HB}\simeq 0.04$, the chronic steady state becomes unstable via a subcritical Hopf bifurcation (which also gives rise to an unstable periodic solution). For $\phi>\phi _{HB}$, the model is monostable and damage is guaranteed to resolve since the only stable configuration is the healthy steady state. In Fig. 3(b), we also show the corresponding levels of ECM for the solutions shown in panel (a), since increased levels of ECM are commonly associated with chronic outcomes. Here, lower levels of macrophage phagocytosis ($\phi $) result in greater numbers of apoptotic neutrophils, and therefore higher levels of pro-inflammatory, more hepatocyte damage and stellate cell activation, and thus greater levels of ECM production via (2i). (Indeed, we can make similar observations as a function of any other parameter that stimulates (or reduces) pro-inflammatory mediator concentrations, and we revisit this with a particular focus on the effect of hepatocyte damage upon ECM production later in this manuscript.) In Fig. 3(c), we hold fixed $\phi =0.1$ and instead vary the rate of neutrophil apoptosis, $\nu $. We observe similar behaviour to Fig. 3(a), with the model being bistable for $\nu <\nu _{HB}\simeq 0.006$, and monostable (guaranteeing resolution) for larger values of $\nu $. In this case, the Hopf bifurcation is supercritical, giving rise to a branch of stable periodic solutions that exist in a narrow window of $\nu $-values, until these are eliminated via a saddle node of periodic orbits (SNPO). In Fig. 3(d), we track the position of this Hopf bifurcation in $(\phi ,\nu )$-space, determining regions of bistability (B) and monostability with guaranteed resolution (M:Res). Intuitively, Fig. 3(d) reveals that for very low rates of neutrophil apoptosis, raising the rate of macrophage phagocytosis is insufficient in eliminating the possibility for chronic outcomes (since, in this case, neutrophils remain in their active state for much longer and hence are not removed by macrophages, which only target apoptotic cells).

### 4.2 Responses to reducing pro-inflammatory mediator production

**Fig. 4.**
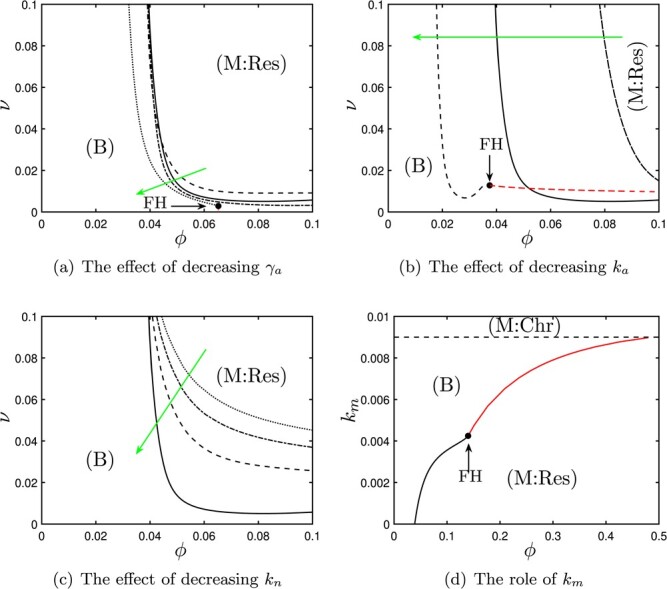
Bifurcation diagrams for the system (2) showing the effect of variations in the strength of pro-inflammatory mediator feedbacks by apoptotic neutrophils ($\gamma _a$, $k_a$), active neutrophils ($k_n$) and $m_i$ macrophages ($k_m$), for the parameters values given in Table 3. In (a–c), green arrows indicate decreases in the parameter of interest. (a) Positions of Hopf bifurcations for $\gamma _a=0.5$ (dashed), $\gamma _a=1$ (solid), $\gamma _a=2$ (dash-dotted), $\gamma _a=10$ (dotted). (b) Positions of Hopf (black) and saddle-node (red) bifurcations for $k_a$ halved/doubled (dash-dotted/dashed respectively) compared with baseline values (solid line). (c) Positions of Hopf bifurcations for $k_n=0.01$ (solid), $k_n=0.04$ (dashed), $k_n=0.07$ (dash-dotted), $k_n=0.1$ (dotted). (d) The effect of $k_m$, shown in $(\phi ,k_m)$–space, showing the positions of Hopf (solid black) and saddle-node (solid red) bifurcations affecting chronic steady states, and the transcritical bifurcation affecting the healthy steady state as per (2) (dashed). (Abbreviations: B indicates bistable; M:Res, monostable (resolution); M:Chr, monostable (chronic); FH, Fold–Hopf bifurcation.)

Here, we investigate the model’s response to reductions in the rates of production of pro-inflammatory mediators, focusing in particular upon production by apoptotic neutrophils (via $\gamma _a$ and dimensional parameter $k_a$), active neutrophils ($k_n$) and $m_i$ macrophages ($k_m$). In Fig. 4, we show bifurcation diagrams that illustrate the effect of varying each of these parameters in turn. The green arrows in Fig. 4(a–c) illustrate how the curves that bound regions of bistability (or equivalently regions of guaranteed resolution of damage) move in $(\phi ,\nu )$-space as each neutrophil-based feedback is reduced, e.g. due to therapeutic intervention.

In Fig. 4(a and b), we examine how reducing the strength of the pro-inflammatory feedback from apoptotic neutrophils shifts the boundary between bistability and resolution. There are two parameters that govern this: $\gamma _a$, which is the rate of neutrophil necrosis; and $k_a$, which is a measure of the amount of pro-inflammatory mediator released on this event. (We note here that the parameter $k_a$ is dimensional and was scaled out under our nondimensionalisation. Hence, it does not appear in (2) directly; investigating the effect of variations in this parameter therefore requires revisiting some of the dimensionless parameter groups given in (3).) In Fig. 4(a), we observe that gradually decreasing $\gamma _a$ has only a relatively marginal effect upon the system’s outcomes, moving the boundary between bistability and resolution slowly to the left in the figure and hence slightly enlarging the region of parameterspace in which resolution is guaranteed. In Fig. 4(b), we instead investigate the effect of manipulating $k_a$. To do this, we start with the baseline set of parameters given in Table 3, represented by the solid line in the figure. To obtain the dash-dotted line in the figure, we then halve $k_a$, which corresponds to reducing the parameters $k_g$, $k_h$, $r_1$ and $\nu _2$ by a corresponding amount and also doubling $\beta _c$, $\beta _a$, $\beta _n$, $\gamma _{m2}$ and $k_n$ (due to (3)). Likewise, to obtain the dashed curve, we double $k_a$ from its original value in Table 3 and make the converse adjustments to the remaining parameters. We then plot the boundary of bistability in $(\phi ,\nu )$-space as before. Note that we here plot $\phi $ on the horizontal axis for clarity of results; however, $\phi $ itself also scales with $k_a$ as per (3a). We observe, here, that reducing the strength of pro-inflammatory mediator production by apoptotic neutrophils via $k_a$ results in an enlargement of the region of guaranteed resolution, as the boundary is shifted left in the figure. We note, however, that the position of this boundary scales roughly linearly with the extent to which $k_a$ is varied. Plotting $\phi /k_a$ on the horizontal axis would yield a figure qualitatively similar to that of panel (a). It is interesting, from a dynamical systems perspective, to note that for the larger choice of $k_a$ (dashed line) and some small values of $\nu $ there exist two Hopf bifurcations that bound a narrow window of oscillations. Additionally, for $\gamma _a=10$ in Fig. 4(a) or for the larger choice of $k_a$ in Fig. 4(b), we observe the tangential collision of a branch of Hopf bifurcations with a branch of saddle-nodes (shown in red). This collision point is a Fold–Hopf (FH) bifurcation and results in the Hopf bifurcation itself being eliminated. The associated dynamics are not particularly significant here, since they involve changes in the number of additional unstable chronic branches. In Fig. 4, we have only plotted the sections of the saddle-node branches that bound our bistability region, for clarity.

In Fig. 4(c), we show the effect of reducing the pro-inflammatory feedback of active neutrophils (with parameter $k_n$). This has a more significant impact upon the observed monostable and bistable regions, with a reduction of $k_n$ driving the boundary between these in the direction of the green arrow shown, with guaranteed resolution of damage in a growing region of parameterspace. In particular, as $k_n$ is reduced, the system is able to guarantee recovery from damage for successively weaker levels of macrophage phagocytic ability ($\phi $).

In Fig. 4(d), we show how the production of pro-inflammatory mediators by macrophages ($k_m$) affects the outcomes of the model. We have already seen, in (5c) above, that large choices of $k_m$ result in the healthy (fully resolved) steady state being unstable. This therefore results in an area of parameterspace (in this case, the region $k_m>0.009$) in which chronic damage is the only permissible outcome. Regardless of $\phi $, reducing $k_m$ through this threshold results in the healthy steady state becoming stable. For $\phi \lesssim 0.48$, the chronic steady state also remains stable initially, resulting in a region of bistability in which the outcome depends upon the magnitude of the damage stimulus. For $\phi \gtrsim 0.48$, the chronic steady state is destabilized immediately as the healthy configuration becomes stable. For all choices of $k_m<0.009$ here, the production of pro-inflammatory mediators via $k_m$ is sufficiently weak that it can be overcome by sufficiently strong macrophage phagocytic ability, yielding a region of guaranteed resolution (M:Res). This region is bounded above by branches of either Hopf or saddle-node bifurcations, which meet tangentially at a FH bifurcation as described above. (Again, we only plot the portions of the saddle-node branches that bound our region of bistability, here, for clarity).

### 4.3 Stimulating production of anti-inflammatory mediators

**Fig. 5.**
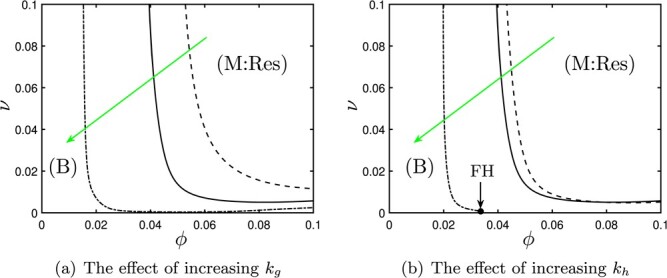
Bifurcation diagrams for the system (2) showing the effect of variations in the strength of anti-inflammatory mediator feedbacks by $m_r$ macrophages ($k_g$) and hepatocytes ($k_h$), for the parameters values given in Table 3. Green arrows indicate increases in the parameter of interest. (a) Positions of Hopf bifurcations for $k_g=0.01$ (dashed), $k_g=0.1$ (solid) and $k_g=1$ (dash-dotted). (b) Positions of Hopf bifurcations for $k_h=0.01$ (dashed), $k_h=0.1$ (solid) and $k_h=1$ (dash-dotted). (Abbreviations: B, bistable; M:Res, monostable (resolution); FH, Fold–Hopf bifurcation.)

In Fig. 5, we illustrate the effect of increasing the levels of anti-inflammatory mediators by macrophages (via $k_g$) and hepatocytes (via $k_h$). Again, we illustrate how the boundary between regions of bistability (B) and guaranteed resolution (M:Res) moves in $(\phi ,\nu )$-space as we vary each of $k_g$ and $k_h$ individually. In Fig. 5, the green arrow indicates the effect of increasing each of these parameters (which is akin to increasing the rates of production of anti-inflammatory mediators). Essentially, the effect of increasing either $k_g$ or $k_h$ is very similar to that observed on decreasing the production of pro-inflammatory mediators above (in Fig. 4), with the boundary of the bistable region begin shifted left (and down) in both panels of Fig. 5. That is, if the levels of anti-inflammatory mediators present in the system are increased, the system can withstand reductions in the phagocytic ability of macrophages ($\phi $), with resolution of damage being guaranteed for smaller values of $\phi $. (Once again, the boundaries of the bistable region are generally branches of Hopf bifurcations, although it is possible in some cases that these branches collide tangentially with branches of saddle-nodes at FH points, as marked by FH in Fig. 5.)

It is perhaps evident, here, that up-regulating anti-inflammatory mediator production by macrophages or hepatocytes has a greater effect on the system than does down-regulating pro-inflammatory mediator production via neutrophils (cf. Fig. 4(a and c)); this is evident in that the shift in the boundary of the bistable region is more substantial in Fig. 5 (for the parameters studied here, at least). It is perhaps pertinent to note that the effects of manipulating pro-inflammatory feedbacks from neutrophils or anti-inflammatory feedbacks from macrophages or hepatocytes are all manifest in changes to the stability of chronic steady state and therefore present switches from monostable (resolved) to bistable only. Manipulation of the pro-inflammatory feedback from $m_i$ macrophages ($k_m$) plays a much stronger role in determining outcomes, however, since this parameter also has an effect upon the stability of the healthy steady state, disruption of which can result in guaranteed chronicity (M:Chr in Fig. 4(d)).

### 4.4 Hepatocyte damage

In this section, we examine the impact that hepatocyte damage (i.e. stimulated hepatocyte apoptosis) has upon the outcomes of our model. The primary parameter of relevance here is $\nu _2$, which captures the susceptibility of hepatocytes to damage. Increasing $\nu _2$ results in an increase in $h_a$ (the number of hepatocytes) which provides a stimulated pro-inflammatory feedback via the third term in the right-hand side of equation (2e). In parallel to this, the corresponding reduction in $h$ (the number of healthy hepatocytes) reduces the strength of the anti-inflammatory feedback given by the second term in the right-hand side of (2f). Intuitively, we thus expect stimulated hepatocyte damage to worsen the inflammatory outcome.

We have already established, in (5c), that $\nu _2$ plays a strong role in determining the permissible long-term outcomes of our model, since variations in this parameter directly impact upon the stability of the healthy steady state. Equation (5c) reflects that the key players in determining, in tandem, whether a resolved outcome is permissible are macrophages and hepatocytes, with associated feedback parameters $k_m$ and $\nu _2$ and decay parameters $\gamma _m$ and $\gamma _h$, respectively. It is the combined contribution of these cell groups that determines whether a healthy (resolved) outcome can present; if the pro-inflammatory contributions of hepatocytes are sufficiently upscaled (via $\nu _2$), then, unless there is a compensating reduction in the macrophage feedback (via $k_m$), the healthy steady state will become unstable and chronic outcomes become certain. (We may also make a similar (converse) argument regarding manipulation of the decay parameters $\gamma _m$ and $\gamma _h$.)

**Fig. 6.**
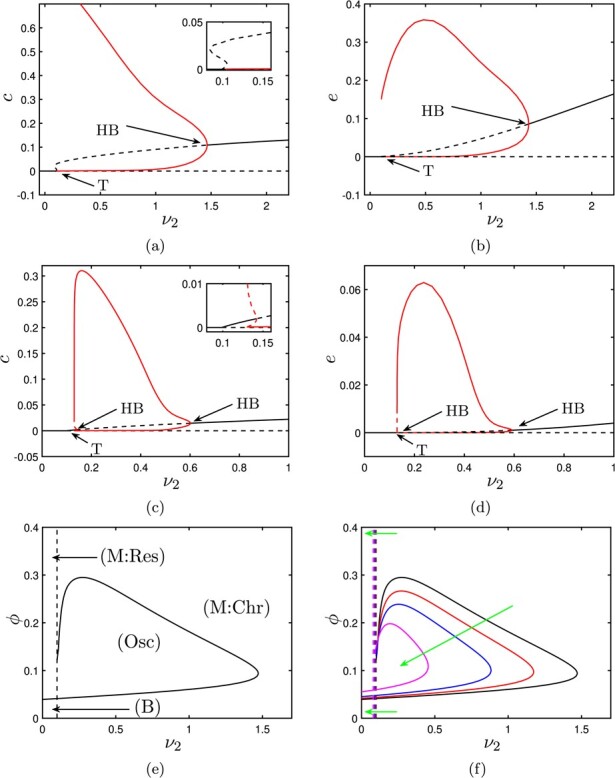
Bifurcation diagrams for the system (2), showing the role of the hepatocyte apoptosis rate parameter $\nu _2$. In (a–d), solid (*resp.* dashed) black lines represent stable (*resp.* unstable) fixed points; solid (*resp.* dashed) red lines represent stable (*resp.* unstable) periodic orbits. (a,b) Bifurcation diagrams for the parameters given in Table 3. For $\nu _2$ large, only the chronic steady state is stable. As $\nu _2$ is decreased, stable oscillations arise via a supercritical Hopf bifurcation (HB). These oscillations grow in amplitude until they collide with the trivial steady state at $\nu _2\simeq 0.1$ and are hence eliminated via a homoclinic bifurcation. Also, for $\nu _2\simeq 0.1$, the trivial steady state undergoes a transcritical bifurcation (T), rendering the system monostable with guaranteed resolution for $\nu _2\lesssim 0.1$. (c,d) For $\phi =0.25$, we see similar behaviour to in (a); however, the oscillations are this time contained between two Hopf bifurcations, the left-most of which being subcritical. In (e), we show the position of the Hopf bifurcation (solid line) and transcritical bifurcation (dashed line) in $(\nu _2,\phi )$–space, identifying two regions of monostability (with resolved or chronic outcomes; M:Res and M:Chr, respectively) and a region of stable oscillations (Osc). (f) The effect of increasing $k_m$, as indicated by the green arrows; higher values of $k_m$ reduce the size of the region of oscillations and shift the transcritical bifurcation to the left. The curves shown are for $k_m=0.0001$ (black), $k_m=0.0005$ (red), $k_m=0.001$ (blue), $k_m=0.002$ (magenta).

**Fig. 7.**
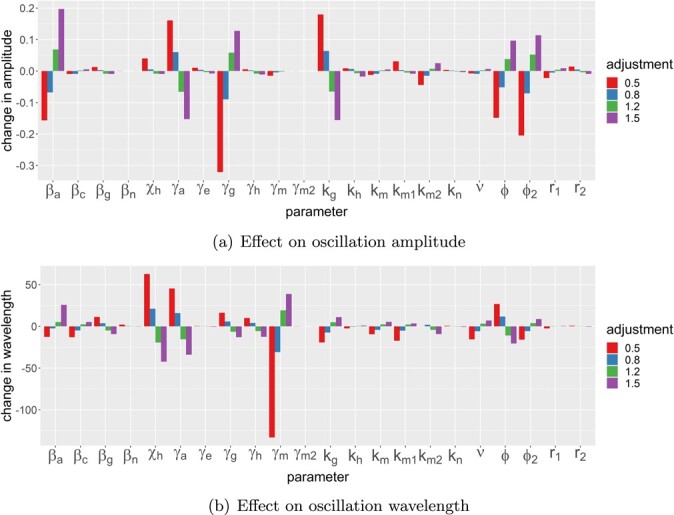
Sensitivity of the wavelength and amplitude (in $c$) of oscillatory solutions to variations in parameters. Parameters are varied (by $\pm 20\%$ or $\pm 50\%$) around the values given in Table 3, with $\nu _2=0.5$. The changes in wavelength and amplitude are absolute (dimensionless) values.

In Fig. 6, we illustrate bifurcation diagrams that show how variations in $\nu _2$ effect not only the stability of the healthy steady state but also the nature of chronic outcomes. In Fig. 6(a), we show a bifurcation diagram that plots the $c$-coordinate of steady states and periodic orbits as a function of $\nu _2$ for the parameter values given in Table 3. As we know from (5c), the healthy steady state loses stability at $\nu _2=0.099$: for $\nu _2<0.099$, the healthy steady state is stable and healthy outcomes are guaranteed; for $\nu _2>0.099$, the healthy steady state is a saddle and only chronic outcomes (either steady state or periodic) are permissible. The branch of chronic steady states undergoes a supercritical Hopf bifurcation at $\nu _2\simeq 1.5$. For $\nu _2\gtrsim 1.5$, a stable chronic steady state exists and is the guaranteed outcome. For $\nu _2\lesssim 1.5$, the chronic steady state is unstable and surrounded by a stable periodic orbit, whose amplitude grows as $\nu _2$ is further decreased, until the periodic orbit eventually collides with a stable manifold of the saddle that represents the healthy steady state; the periodic orbit is there eliminated via a homoclinic bifurcation. Figure 6(b) shows the levels of ECM corresponding to the branches of Fig. 6(a), with large values of $\nu _2$ resulting in chronic steady states with ECM levels elevated by approximately 10%–20%. (These values are broadly consistent with the experimental observations reported in Bedossa & Paradis, 2003, which suggest that fibrotic liver tissue can be comprised of approximately 15% ECM.) In Fig. 6(c), we show a similar bifurcation diagram, but for $\phi =0.25$ (and all other parameters as in Table 3). We once again observe switching between healthy steady states, chronic steady states and oscillatory solutions; however, for these parameters, the oscillations lie between two Hopf bifurcations, rather than being eliminated via collision with the healthy steady state as in Fig. 6(a). Figure 6(d) illustrates corresponding ECM levels; as we have already observed in Fig. 3(c), larger values of $\phi $ seem to correlate with lower levels of ECM deposition. As shown in Fig. 6(e), the branch of Hopf bifurcations can be traced in $(\nu _2,\phi )$-space and, together with the transcritical bifurcation of (5c), bounds a self-contained region of parameterspace in which oscillatory solutions exist. The size of this region of oscillations scales inversely with $k_m$, as shown in Fig. 6(f); as $k_m$ is increased, the transcritical bifurcation of (5c) moves left in the figure, while the region of oscillations decreases in size. For sufficiently large increases in $k_m$, beyond the point marked FH in Fig. 4(d), oscillations are eliminated entirely as the corresponding Hopf bifurcation collapses onto a branch of saddle nodes, as per Fig. 4(d). The amplitude and wavelength of these oscillatory solutions can be controlled by varying the remaining model parameters (as shown for $\nu _2=0.5$ in Fig. 7). For the parameters studied here, it seems that the amplitude of these oscillations is predominantly controlled by a balance between the strength of pro-inflammatory feedbacks from neutrophils ($\beta _a$, $\gamma _a$), the negative feedbacks of macrophages via anti-inflammatory mediators ($k_g$) and the ability of macrophages to remove apoptotic neutrophils via phagocytosis ($\phi $, $\phi _2$). Conversely, the wavelength of these solutions is most strongly affected by the necrosis ($\gamma _a$) and macrophage decay ($\gamma _h$) parameters, as well as $\chi _h$, which is related to the relative ability of macrophages to remove damaged hepatocytes, compared with apoptotic neutrophils. It appears, therefore, that hepatocyte damage plays a strong role in the existence of oscillatory solutions via $\nu _2$, but the nature of these oscillations themselves are predominantly controlled by mechanisms related to macrophage–neutrophil interactions.

## 5. Conclusion

Hepatitis is associated with ongoing inflammation, but the inflammatory infiltrate is normally short lived and resolves without impacting liver tissue. If inflammation is allowed to persist, then scar tissue is formed that can progress to cirrhosis characterized by a loss of liver function and can ultimately lead to liver failure.

We have developed a model of hepatitis that captures the interactions between the liver’s key cell types and the acute immune response. The model has two fundamental steady states, representing disparate outcomes. Firstly, there exists a trivial solution, where after a stimulus the inflammatory components settle to zero and the cells of the liver return to their original positive values. Secondly, for some parameters, there may be a second steady state where the inflammatory components settle to a positive value and the liver’s key cell types fail to return to healthy values. We equate these steady states to healthy resolution and ongoing, self-perpetuating liver damage, respectively. Depending on our choice of parameters values, our model may exhibit guaranteed resolution of damage, with only the resolved state stable; guaranteed chronicity, with the resolved state being unstable; or the model may be bistable with the outcome attained being determined by the severity of the damage stimulus. We have explored the effects that perturbation of parameters has on model outcomes via numerical simulation and bifurcation analysis and used the model to identify how individual parameters govern the behaviour of the system.

We found that bistability and hysteresis arise in large regions of parameter space. In Fig. 3, we investigated the role of two key mechanisms known for their variability under the influence of inflammatory conditions on model outcomes; these are the rate that macrophages remove apoptotic neutrophils ($\phi $) and the rate that neutrophils die via apoptosis ($\nu $). As in previous work on generic inflammation, we found that when macrophages are inefficient at removing dead cells the model is bistable but increasing macrophage efficiency ensures resolution to a healthy outcome. This supports recent work that investigates the use of resolvins and protectins, a class of autacoids that can increase rates of macrophage phagocytosis, as therapeutics for non-alcoholic fatty liver disease (Remien *et al.*, 2012). Neutrophils release toxic contents both when active and apoptotic, the latter occurring under necrosis. This dichotomy makes it unclear intuitively if neutrophil apoptosis has a pro- or anti-inflammatory effect. Here, under low levels of neutrophil apoptosis the system is bistable but again shifts to a monostable outcome, that ensures resolution, at higher values. The dependence between neutrophil apoptosis and macrophage phagocytosis was shown and highlights that the anti-inflammatory effect of macrophages is neutralized under low levels of neutrophil apoptosis, a situation known to occur in inflammatory conditions.

In Fig. 4, we investigated the influence of the many different sources of pro-inflammatory mediators on regions of bistability and resolution. We found that the ability to down-regulate any one mechanism increases regions of resolution. But, we found that controlling the production of pro-inflammatory mediators from macrophages was particularly important. Excess production of these mediators from macrophages shifts the model into regions of parameterspace where self-perpetuating liver disease is guaranteed, highlighting the therapeutic benefit of targeting this mechanism, which is under investigation for the treatment of acute liver failure (Wang *et al.*, 2021). The therapeutic use of macrophages modified *in vitro* to generate proresolution features has been postulated for fibrotic liver disease (Pellicoro *et al.*, 2014). Interestingly, our model indicates that stimulation of anti-inflammatory mediator production via restorative macrophages ($k_g$) constitutes one effective switch to promote guaranteed resolution of damage (see Fig. 5(a)); this is in direct agreement with the conclusions of Pellicoro *et al.* (2014).

Damage to hepatocytes, which comprise 80% of a healthy liver, occurs not only under rising levels inflammatory mediators but as a direct result of the toxicity of some chemicals and medications, such as acetaminophen (paracetamol), a common cause of liver failure. As such, methods to protect hepatocytes from damage is under multiple investigations (Koyama & Brenner, 2017; Boland *et al.*, 2020). While our current model simplifies hepatocyte damage to a single inflammatory mechanism we showed, in Fig. 6, the effect of variation in this mechanisms finding that it, alongside macrophage function, is key to determining if resolution of inflammation, and hence liver damage, is possible.

The emergence of oscillatory dynamics is of interest, not only from a mathematical perspective but also biologically in the ongoing search for interventions. For example, oscillations in serum ferritin (a marker of a chronic inflammatory state) are associated with antiviral therapy in chronic hepatitis C (Kronborg *et al.*, 2021); oscillations in interleukin IL-2 (a pro-inflammatory mediator) are reported in adaptive strategies for IL-2 therapies (which are of relevance to hepatitis treatment) (Ju & Tacke, 2016); and (although not explicitly modelled here), oscillatory dynamics in T cell functions have been associated with hepatitis B virus (Hilscher & Shah, 2020). Our analyses have illustrated (in Section 4.4 in particular) that this model emits oscillations for a wider range of parameter values than does the previous model of generic inflammation of Dunster *et al.* (2014), with the rate of hepatocyte damage ($\nu _2$) being the parameter that (in tandem with the macrophage phagocytosis parameter, $\phi $) exhibits the strongest influence over the existence of oscillations (as illustrated in Fig. 6). Furthermore, in $(\nu _2,\phi )$-space, the size of the region of oscillatory solutions scales inversely with the rate that macrophages produce pro-inflammatory mediators, $k_m$. It is notable that, while the existence of oscillatory solutions is strongly linked to the scale of hepatocyte damage ($\nu _2$), the scale (amplitude) and timing (wavelength) of these oscillations seems to be predominantly governed by mechanisms related to macrophage–neutrophil interactions (as shown in Fig. 7).

There are many ways in which our mathematical model could be extended and improved going forward. In the model presented here, we adopt a relatively simplistic description of the very diverse range of cell types and mediators that play a role in maintaining liver health. For example, while our model includes two opposing macrophage phenotypes (which may broadly be regarded as representative of entirely pro- or anti-inflammatory populations), in reality the range of macrophage phenotypes is known to be much broader than this (Dunster, 2016). While the model could be enhanced to account for more macrophage phenotypes (or perhaps a continuous spectrum of behaviours), we note that this would present numerous challenges with regard to accurate parameterization of the model—a task which is already relatively complex under our current two-phenotype description. Our model also neglects other cell types that undoubtedly play a role in disease progression, e.g. T cells and platelets. Platelets, in particular, are known to effect multiple (and often contradicting) mechanisms that underlie hepatitis (Chauhan *et al.*, 2016; van der Heide *et al.*, 2019). They can increase migration of cells, such as neutrophils, into the liver thus amplifying liver damage, and modify the hepatic cellular and cytokine milieu driving both hepatoprotective and hepatotoxic processes (Chauhan *et al.*, 2016). Platelets from different donors can be stratified into a range of functional phenotypes that could well play a role in determining disease progression (Dunster *et al.*, 2021). Investigation of how these divergent platelet effects impact upon the dynamics of our model remains one of our primary targets for future work. Our model also neglects explicit descriptions of individual cytokines such as interleukins IL–2, IL–10 and IL–12 and IFN–$\gamma $, instead grouping these into generic mediators. Our analysis here has focussed on the outcomes of hepatitis (i.e. the switch between resolution, chronic damage and oscillatory outcomes); however, it is pertinent to note that dynamical models can also offer insight into the timing of events that are often hard to observe experimentally. Experimental data to date have shown that a typical inflammatory timecourse comprises an early peak in neutrophil numbers, followed by a peak in inflammatory macrophages, and later followed by peaks in restorative macrophages (Tang *et al.*, 2021). While our simulations show neutrophil peaks preceding those of macrophages, as expected, some of our simulations predicted restorative macrophage phenotypes reaching peak levels slightly before pro-inflammatory phenotypes, which contradicts current evidence. Investigation of this particular facet of the model indicate that the timing of these macrophage peaks can be manipulated by modification of parameter values, with saturation constants, in particular, seeming to play a key role. However, since performing this type of model tuning in a meaningful manner would require a greater quality of parameterization data than is currently available, this has not been a focus of our current analysis and remains one target for future consideration. While the simplicity of our model allowed us to reduce the model’s parameters and focus on what are thought to be the dominant mechanisms, in order to facilitate analytical progress, the model could be easily extended to incorporate more detailed descriptions of these aspects as corresponding clinical or research data become available. Finally, our model neglects a spatial description of the liver’s structure and hence does not account for positions of key cell types and geometric aspects of cell recruitment and mediator spreading. A natural extension to our model would be to incorporate these spatial descriptions. This could involve moving to either a PDE or agent-based modelling approach, both of which have been previously considered in a more generic inflammatory setting by e.g. Bayani *et al.* (2020a,b). The agent-based approach is particularly favourable here, as it has previously been shown to provide more realistic descriptions of chemotactic migration by immune cells, and the role this plays in resolving inflammatory damage Bayani *et al.* (2020b). Again, this remains a target for future study.

Given that there are currently no disease-modifying treatments for hepatitis and that there is a significant lack of sufficient liver biopsy samples, mouse models and biomarkers to provide data on the progression of the disease and the state of inflammation in the liver (Lauffenburger & Kennedy, 1983), we conjecture that mathematical and computational models of hepatitis can play a pivotal role in understanding the complex mechanisms that govern liver disease. Going forward, mathematical models such as that presented here offer great scope in addressing questions around which mechanisms are the optimal targets for therapeutic interventions, and also how best to strategize dosing frequencies and scales, for instance. While we have begun to address some of these questions here, there is a great deal of opportunity to further develop this model and others in the future, with scope for model development to guide the collection of experimental and clinical data, with these data influencing model design, parameterization and validation in tandem.
